# Settingübergreifende Behandlung in der Psychiatrie: Umsetzung spezifischer Versorgungsmerkmale an Kliniken der Modell- und Regelversorgung (PsychCare-Studie)

**DOI:** 10.1007/s00115-021-01238-2

**Published:** 2021-12-07

**Authors:** Julian Schwarz, Yuriy Ignatyev, Fabian Baum, Anne Neumann, Bettina Soltmann, Andrea Pfennig, Jürgen Timm, Martin Heinze, Sebastian von Peter

**Affiliations:** 1 Immanuel Klinik Rüdersdorf, Hochschulklinik für Psychiatrie und Psychotherapie der Medizinischen Hochschule Brandenburg, Seebad 82/83, 15562 Rüdersdorf, Deutschland; 2 Zentrum für Versorgungsforschung Brandenburg, Medizinische Hochschule Brandenburg, Neuruppin, Deutschland; 3 Zentrum für Evidenzbasierte Gesundheitsversorgung, Universitätsklinikum Carl Gustav Carus an der Technischen Universität Dresden, Dresden, Deutschland; 4 Klinik und Poliklinik für Psychiatrie und Psychotherapie, Universitätsklinikum Carl Gustav Carus, Medizinische Fakultät, Technische Universität Dresden, Dresden, Deutschland; 5 Kompetenzzentrum für Klinische Studien Bremen, Universität Bremen, Bremen, Deutschland

**Keywords:** Globalbudget, Sektorenübergreifend, Integrierte Versorgung, Psychiatrische Versorgung, Versorgungsqualität, Qualitätsindikatoren, Implementierung, Global budget, Cross-sectoral, Integrated care, Mental health care, Health care quality, Quality indicators, Implementation

## Abstract

**Hintergrund:**

Seit 2003 wird an einzelnen psychiatrischen Kliniken ein neues Vergütungs- und Versorgungsmodell erprobt, welches auf Grundlage eines globalen Behandlungsbudgets eine settingübergreifende, integrative sowie Zuhausebehandlung bietet. Derzeit existieren bundesweit 22 dieser psychiatrischen Modellvorhaben nach § 64b SGB V (MV). Die bisherige Forschung konnte 11 spezifische Struktur- und Prozessmerkmale zur Einstufung von MV identifizieren, die allerdings noch nicht in einer kontrollierten Studie hinsichtlich ihrer methodischen Anwendbarkeit überprüft wurden. Untersucht wird die statistische Unterscheidungsfähigkeit der Merkmale an Kliniken der Regel- und der Modellversorgung.

**Methode:**

Als Teil der PsychCare-Studie wurde an 9 Modell- und 7 Kontrollkliniken die Einstufung der Merkmale vorgenommen und vergleichend sowie in Subgruppen analysiert. Die Subgruppen unterteilen jene Kliniken, die mit allen oder nur einem Teil der Krankenkassen ein MV vereinbart haben.

**Ergebnisse:**

Sieben der 11 Merkmale (Flexibilität im Settingwechsel, settingübergreifende therapeutische Gruppen, Zuhausebehandlung, systematischer Einbezug von Bezugspersonen, Erreichbarkeit von Leistungen, sektorübergreifende Kooperation und Erweiterung professioneller Expertise) wiesen eine hohe statistische Unterscheidungsfähigkeit auf. In den Subgruppen waren diese Unterschiede tendenziell stärker ausgeprägt.

**Schlussfolgerung:**

Die modellspezifischen Merkmale sind geeignet, um Qualitätsunterschiede der Implementierung settingübergreifender, flexibler und aufsuchender Versorgung zu evaluieren.

## Abstract (en)

**Background:**

Since 2003 a new remuneration and care model has been implemented in currently 22 psychiatric clinics in Germany, which provides flexible and integrative treatment (FIT) across different settings of treatment, including assertive outreach care. The FIT is based on a global treatment budget covering costs for all psychiatric hospital services and is related to the number of patients treated per year. Previous research identified 11 program components that describe treatment structures and processes of FIT. The present analysis quantitatively compared FIT with standard care, aiming to validate these components.

**Methods:**

As part of the PsychCare study, grading according to FIT-specific components was carried out and comparatively analyzed in nine hospitals that used FIT and seven hospitals of standard care. The FIT models were examined in depth in subgroups, which were divided according to the share of the FIT model in the total hospital budget.

**Results:**

Of the 11 FIT program components 7 (flexible care management across settings, therapeutic group sessions across all settings, outreach home care, involvement of informal caregivers, accessibility of services, cooperation across sectors and expansion of professional expertise) showed a high statistical differentiation. These differences were even stronger in the subgroup of those hospitals that had a model contract with all health insurers.

**Conclusion:**

Specific components of FIT are suitable for evaluating the quality of implementation of intersectoral, flexible and outreach psychiatric care.

## Hintergrund

Seit 2003 wird an ausgewählten psychiatrischen Kliniken Deutschlands eine settingübergreifende Behandlung, einschließlich einer akutambulanten und aufsuchenden Versorgung im häuslichen Umfeld erprobt [1–3]. Bisher haben 22 Fachkliniken und Abteilungen an Allgemeinkrankenhäusern ein sog. Modellvorhaben nach § 64b SGB V (MV^1^) – so der aktuelle gesetzliche Rahmen dieser Versorgungsform – eingeführt. Finanzielle Grundlage der MV ist ein globales Behandlungsbudget (GBB), welches mit den Krankenkassen für eine Laufzeit von bis zu 15 Jahren vereinbart wird. Entweder können GBB als Selektivvertrag mit einzelnen Krankenkassen oder als regionales Psychiatriebudget (RPB) mit allen in einer Region vertretenen Krankenkassen verhandelt werden [4]. Abweichend von der sonst üblichen tages- und leistungsbezogenen Vergütung gemäß dem Pauschalierenden Entgeltsystem für Psychiatrie und Psychosomatik (PEPP), richten sich GBBs nach der Anzahl der in einem Jahr von einer Klinik behandelten Patienten [5].

Erste Ergebnisse der gesetzlich verpflichtenden Evaluation (nach § 65 SGB V; „EVA64“-Studie) weisen auf eine teilweise Reduktion der stationären Behandlungstage in den MV hin [6–8]. Durch in einzelnen Modellregionen durchgeführte Begleitforschung konnte außerdem eine Reduktion von Betten, der voll- und teilstationären Belegungstage und der kumulativen Verweildauer belegt werden [9–11]. Zugleich wurde eine Verringerung von Krankheitsschwere, eine Verbesserung des psychosozialen Funktionsniveaus, der subjektiver Lebensqualität und der Behandlungskontinuität bei gleichzeitiger Reduktion der Gesamtkosten der psychiatrischen Versorgung nachgewiesen [1, 9–11].

In einer multizentrischen Prozessevaluation („EvaMod64b“-Studie) konnten spezifische Implementierungsmerkmale settingübergreifender Behandlung identifiziert und deren Umsetzungsgrad in den bestehenden MV ermittelt werden [12–17]. Die Merkmale, welche die Strukturen und Prozesse der MV erfassen, wurden zu Evaluationszwecken entwickelt und sollen zur Qualitätsentwicklung der psychiatrischen Versorgung in Deutschland beitragen. Dies umfasst eine mehrstufige Weiterentwicklung der Merkmale hin zu Qualitätsindikatoren, wobei sie insbesondere in Bezug auf ihre Gütekriterien zu überprüfen sind. Es wurde bereits eine hohe Reliabilität sowie Augenschein- und Inhaltsvalidität der Merkmale nachgewiesen [13]. Eines der wichtigsten Gütekriterien von Qualitätsindikatoren stellt die statistische Unterscheidungsfähigkeit dar. Sie beschreibt die Fähigkeit, Merkmalsunterschiede (Variabilität) zwischen verschiedenen Einrichtungen (Diskriminationsfähigkeit) oder im Zeitverlauf (Änderungssensitivität) statistisch nachzuweisen [18].

Ziel der vorliegenden explorativen Analyse ist es, zur Beurteilung der Qualität der modellspezifischen Merkmale beizutragen. Es wird untersucht, ob die Merkmale eine ausreichende statistische Unterscheidungsfähigkeit aufweisen und somit z. B. für die Untersuchung der Unterschiede zwischen Modell- und Regelversorgung geeignet sind.

## Material und Methoden

### Studiendesign

Die vorliegende Untersuchung ist Teil der „PsychCare“-Studie (Laufzeit: 07/2017 bis 06/2021; [19]). PsychCare ist eine prospektive, multizentrische Beobachtungsstudie zur Evaluation der Wirksamkeit, Kosten und Implementierung der Modell- im Vergleich zur Regelversorgung. Die Studie wird gefördert vom Innovationsfond. Sie verfolgt damit das Ziel, Versorgung durch Erkenntnisse aus der Versorgungsforschung längerfristig zu verbessern und einen Transfer neuer Versorgungsformen in die Regelversorgung zu unterstützen [20].

Die hier untersuchten Struktur- und Prozessmerkmale sind von sog. „erlebensbezogenen Merkmalen für eine gute psychiatrische Versorgung“ abzugrenzen, welche ebenfalls im Rahmen dieser Studie entwickelt wurden [21, 22].

Während die Einstufung der Merkmale bisher nur an MV vorgenommen wurde [12], erlaubt das Kontrollgruppendesign der PsychCare-Studie eine vergleichende, explorative Analyse des Vorkommens der Merkmale sowohl an Kliniken der Modell- als auch der Regelversorgung. Die vorliegende Arbeit untersucht die statistische Unterscheidungsfähigkeit der Merkmale quantitativ, in Anlehnung an das Konzept von McGlynn [18]. Aufgrund von Hinweisen aus Vorstudien [15, 17], wonach der Umsetzungsgrad der Merkmale je nach Ausmaß der Krankenkassenbeteiligung am MV variiert, wurden die MV zusätzlich in Subgruppen mit unterschiedlichem Umfang des Modellvertrags analysiert. Datengrundlage der vorliegenden Arbeit bilden 1. die Einstufung der Merkmale sämtlicher Studienzentren sowie 2. deren Struktur- und Leistungsdaten, welche im Rahmen der Baseline-Erhebung der PsychCare-Studie erhoben wurden.

### Sampling

Von den zu Studienbeginn in Deutschland bestehenden 19 MV wurden insgesamt 10 Kliniken der Erwachsenenpsychiatrie sowie 2 Kinder- und Jugendpsychiatrien mittels einer geschichteten Zufallsziehung ausgewählt. Aufgrund von gegenüber der Erwachsenenpsychiatrie abweichenden Strukturmerkmalen wurden die kinder- und jugendpsychiatrischen Kliniken aus der vorliegenden Analyse ausgenommen. Um die größtmögliche Vergleichbarkeit der Kliniken mit Regel- und Modellversorgung zu gewährleisten, wurden die Kontrollkliniken nach einem etablierten Matching-Algorithmus selektiert [23]. Von den 20 ausgewählten Studienkliniken nahmen 16 an der Studie teil, davon 9 MV sowie 7 Kontrollen. Die Studienkliniken befinden sich in Berlin (Charlottenburg-Wilmersdorf, Friedrichshain-Kreuzberg, Neukölln), Hessen (Herborn, Riedstadt), Niedersachsen (Göttingen, Lüneburg), Nordrhein-Westfalen (Bochum, Bonn, Köln), Sachsen (Glauchau, Radebeul) und Schleswig-Holstein (Heide, Heiligenhafen, Itzehoe, Kiel).

### Datenerhebung und -auswertung

#### Implementierungsgrad modellspezifischer Merkmale

Grundlage der Ermittlung des Implementierungsgrades bilden die 11 spezifischen Merkmale der MV [12]. Sie wurden in einem mehrstufigen Prozess auf Basis empirisch-qualitativer Erhebungen in bestehenden MV operationalisiert, gewichtet, quantifiziert und vorläufig validiert, um den Erfüllungsgrad spezifischer Strukturen und Prozesse in konkreten MV beurteilen zu können [12, 13]. Zusammengenommen bilden die Merkmale ein Set grundlegender Qualitätskriterien settingübergreifender, flexibler und integrativer Versorgung.

Die Einschätzung erfolgt jeweils durch Experten (in der Regel die ärztliche Leitung) im Rahmen eines strukturierten Interviews unter Einbezug von Leistungs- und Strukturdaten der jeweiligen Klinik. Jedes Merkmal setzt sich aus 1 bis 4 Items zusammen, die je nach Ausprägung und Gewichtung mit 0–2 Punkten bewertet werden (für Einzelheiten vgl. [13]). Hieraus werden ein merkmalsbezogener sowie ein Gesamtscore gebildet. Letzterer entspricht dem Implementierungsgrad sämtlicher Modellmerkmale an einer Klinik.

Das Merkmal *Ambulantisierung* konnte nicht berücksichtigt werden, da es zwischen den MV sehr unterschiedlich umgesetzt wird und in Folge nicht ausreichend übereinstimmende Parameter für dessen Quantifizierung zur Verfügung stehen [12].

#### Statistik

Die Unterschiede zwischen den Modell- und den Kontrollkliniken wurden mit dem Mann-Whitney-Test bivariat explorativ getestet. Das Signifikanzniveau wurde auf 5 % festgelegt. Die Effektstärke wurde als Cohens d berechnet. Nach Cohen bedeutet ein d zwischen 0,2 und 0,5 einen kleinen Effekt, zwischen 0,5 und 0,8 einen mittleren und ein d > 0,8 einen starken Effekt [24]. In einer vertiefenden Analyse wurden die MV je nach Umfang der Krankenkassenbeteiligung am Modellvertrag in Subgruppen eingeteilt und mit der Kontrollgruppe verglichen. In Anlehnung an Ergebnisse aus Vorstudien wurden die Grenzen der Subgruppen mit < 30 %, 30–90 % und > 90 % Vertragsumfang festgelegt [15, 17]. Die Analysen wurden mit SYSTAT Version 13 (Systat Software Inc., USA) durchgeführt.

#### Struktur- und Leistungsdaten der Studienkliniken

Ergänzend wurden für das Jahr 2019 strukturelle und statistische Daten der Studienkliniken erhoben, um einen umfassenden Vergleich der Gruppe der Modell- und Kontrollkliniken zu ermöglichen. Abgefragt wurden u. a. Informationen zur Trägerschaft, Belegungskapazitäten und durchschnittlicher Verweildauer. Die gelieferten Leistungsdaten wurden auf Basis von Routinedaten (nach § 21 KHG) ermittelt und von den Zentren kumuliert zur Verfügung gestellt.

## Ergebnisse

### Struktur- und Leistungsdaten der Studienkliniken

Die Struktur- und Leistungsdaten aller teilnehmenden Zentren im Gruppenvergleich sind Tab. 1 zu entnehmen. Die Strukturdaten weisen v. a. Auffälligkeiten bei den Angaben zur Trägerschaft und den Behandlungsplätzen auf: Die MV befinden sich ausschließlich in öffentlicher und freigemeinnütziger Trägerschaft, die Kontrollkliniken werden teilweise von privat-gewerblichen Trägern unterhalten. Die mittlere Bettenzahl pro 1000 Einwohner ist in den Kontrollkliniken fast doppelt so hoch wie in den MV. Die Zahl der Tagesklinikplätze liegt in den MV hingegen deutlich höher als in der Kontrollgruppe. Bei den Leistungsdaten zeigt sich in den MV eine geringere Inanspruchnahme des stationären und eine größere Nutzung der übrigen Settings (Tagesklinik, Ambulanz und Zuhausebehandlung). Die Verweildauer von Patienten im vollstationären Setting lag in den Kontrollkliniken etwas höher als in den MV.

**Table 1:** 

Parameter	Modell (*n* = 9)	Vergleich (*n* = 7)
**Strukturdaten**		
*Vertragsumfang des Modellvorhabens (%)*^*a*^		
< 30 %	2	Nicht zutreffend
30 bis 90 %	3	
> 90 %	4	
*Kliniktyp*		
Abteilung an Allgemeinkrankenhaus	4	2
Fachkrankenhaus	4	2
Universitätsklinik	1	3
*Trägerschaft*		
Öffentlich	8	4
Freigemeinnützig	1	1
Privat	0	2
*Bevölkerungsdichte im Einzugsgebiet (Einwohner/km²)*		
Ländlich (< 200)	3	1
Suburban (200–2000)	3	3
Urban (> 2000)	3	3
*Einwohner in der Versorgungsregion*		
< 150.000	3	3
150.000–200.000	1	2
> 200.000	5	2
*Tagesklinische Behandlungsplätze (pro 1000 Einwohner; SD)*^*b*^	0,24 (0,08)	0,16 (0,19)
*Krankenhausbetten (pro 1000 Einwohner; SD)*^*b*^	0,48 (0,09)	0,80 (0,14)
**Leistungsdaten**		
*Inanspruchnahme eines Settings in %, (SD)*^*c,d,e*^		
Station	34,04 (16,43)	51,64 (32,09)
Tagesklinik	9,41 (4,94)	6,74 (3,57)
Ambulanz	75,70 (17,93)	54,65 (22,71)
Zuhausebehandlung	8,78 (14,87)	0,28 (0,00)
*Inanspruchnahme von 2 bzw. 3 Settings in %, (SD)*^*c,d*^		
Ambulanz + Tagesklinik	5,82 (4,97)	2,15 (0,83)
Ambulanz + Tagesklinik + Station	3,36 (3,50)	1,26 (0,44)
*Fälle pro Patient (SD)*^*d*^		
Station	1,42 (0,25)	1,23 (0,20)
Tagesklinik	1,25 (0,70)	1,12 (0,08)
*Verweildauer in Tagen pro Fall, (SD)*^*f*^		
Station	22,10 (4,59)	25,38 (1,93)
Tagesklinik	33,44 (4,91)	29,59 (7,12)

*SD* Standardabweichung

^a^ Vertragsumfang des Modellvorhabens, angegeben als Anteil des Klinikbudgets (in %), welcher als Modellvorhaben verhandelt wurde. Bei den Kontrollkliniken ohne Modellvorhaben wurde „nicht zutreffend“ angegeben

^b^ Mittelwert und Standardabweichung, berechnet aus den Angaben der einzelnen Zentren der Gruppe der Modell- oder Kontrollkliniken

^c^ Ermittelt auf Grundlage der Patientenzahlen pro Setting und Zentrum

^d^ Gewichtet über die Patientenzahlen pro Zentrum

^e^ Durch Mehrfachangaben erhöht sich die Summe der einzelnen Parameter auf über 100 %

^f^ Gewichtet über die Fallzahlen pro Zentrum

### Implementierungsgrad modellspezifischer Merkmale

**Figure 1:**
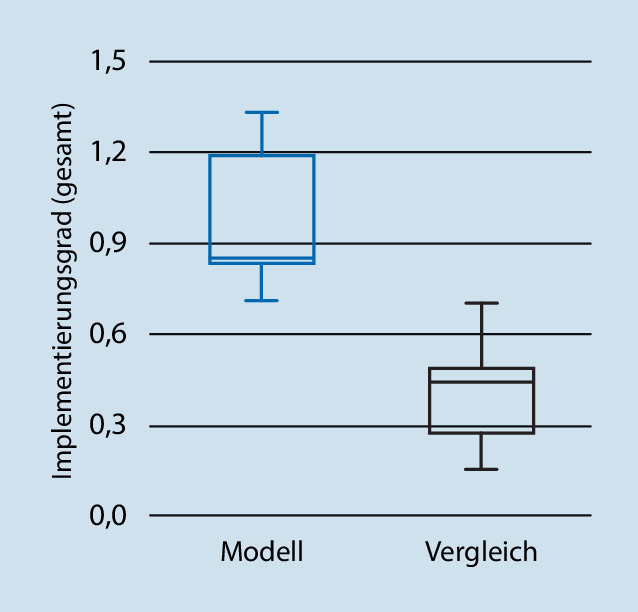

Mit einem Mittel von 0,976 (Gesamtscore) für den Implementierungsgrad lagen die MV mehr als doppelt so hoch wie die Kontrollkliniken mit 0,409 (gesamt). Das Ergebnis ist in Abb. 1 als Box-Plot dargestellt. Der Unterschied ist signifikant (*p* < 0,001) und zeigte eine hohe Effektstärke (Cohens d = 2,61).

Bei Betrachtung des Umsetzungsgrades einzelner Merkmale (Tab. 2) zeigt sich, dass die MV durchweg höhere Mittelwerte für die Merkmale aufweisen als die Kontrollkliniken. Mit Ausnahme der *Merkmale Behandlungskontinuität* (III), *berufsgruppenübergreifende Zusammenarbeit* (IV) und *freie Steuerung therapeutischer Maßnahmen* (IX) sind alle Unterschiede signifikant. Die Effektstärken sind als hoch einzustufen.

**Table 2:** 

Merkmal		Modell	Vergleich	MW-Test	
Nr.	Kurzbeschreibung	M (SD)	M (SD)	*p*	Cohens d
II	Flexibilität im Settingwechsel	2,39 (1,01)	0,85 (0,87)	**0,010**	**1,62**
III	Behandlerkontinuität	0,61 (0,35)	0,13 (0,13)	0,854	1,74
IV	Berufsgruppenübergreifende Zusammenarbeit	2,10 (1,00)	1,54 (0,58)	0,223	0,67
V	Settingübergreifende therapeutische Gruppen	2,00 (0,00)	1,00 (0,76)	**0,001**	**2,00**
VI	Zuhausebehandlung	0,89 (0,33)	0,29 (0,49)	**0,017**	**1,48**
VII	Systematischer Einbezug von Bezugspersonen	0,67 (0,49)	0,19 (0,18)	**0,011**	**1,24**
VIII	Erreichbarkeit von Leistungen	0,85 (0,17)	0,55 (0,18)	**0,004**	**1,76**
IX	Freie Steuerung therapeutischer Maßnahmen	0,68 (0,24)	0,37 (0,25)	0,087	1,23
X	Sektorübergreifende Kooperation	0,71 (0,39)	0,29 (0,24)	**0,031**	**1,26**
XI	Erweiterung der professionellen Expertise	0,63 (0,20)	0,14 (0,13)	**0,011**	**3,15**

Statistisch signifikante Werte sind **fett** hervorgehoben. Die Operationalisierung der einzelnen Merkmale ist dem Beitrag von Peter et al. 2018 zu entnehmen [12]

*Cohens d* Maß für die Effektstärke, *M* Mittelwert, *MW-Test* Mann-Whitney-Test, *p* Signifikanzwert, *SD* Standardabweichung

### Implementierungsgrad im Subgruppenvergleich

**Figure 2:**
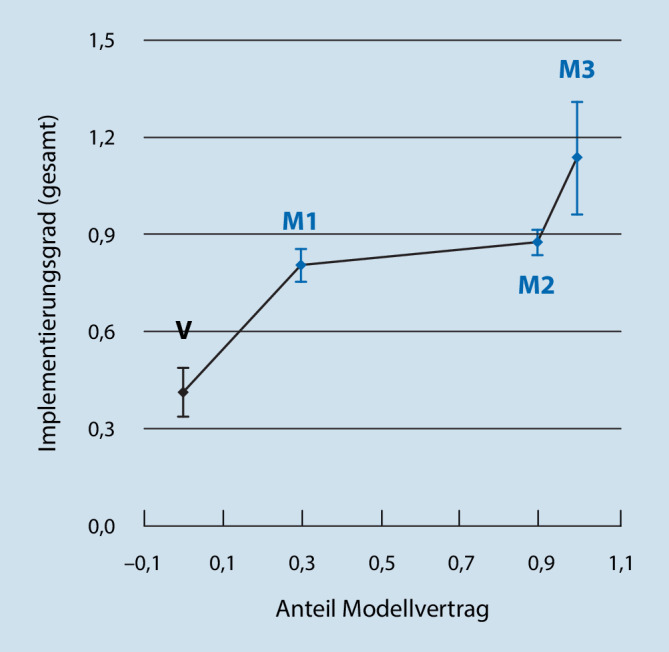

Bei der Untersuchung des Implementierungsgrades (gesamt) der MV im Subgruppenvergleich wurde mit zunehmendem Umfang des Modellvertrags ein höherer Wert erzielt. Das Ergebnis ist in Abb. 2; Tab. 3 dargestellt. Die Unterschiede der Implementierungsgrade (gesamt) zwischen den Kontrollkliniken und den Subgruppen sind für jede Subgruppe signifikant und zeigen eine hohe Effektstärke mit Tendenz zu einem Anstieg der Effektstärke bei zunehmendem Umfang des Modellvertrags (Abb. 2; Tab. 3).

**Table 3:** 

Subgruppe	V	M1	M2	M3
Anzahl Kliniken	7	2	3	4
Anteil Modellvertrag	0	< 0,3	0,3–0,9	> 0,9
*p*	–	0,040	0,016	0,008
Cohens d	–	2,31	2,88	3,22

*V *Gruppe der Kontrollkliniken ohne Modellvertrag, *M1–3* Subgruppen der Modellkliniken mit zunehmendem Vertragsumfang des Modellvorhabens, *p* Signifikanzwert

## Diskussion

In der Zusammenschau der Ergebnisse stellen sich zahlreiche Unterschiede zwischen den untersuchten Studienkliniken der Modell- und der Regelversorgung dar. Das Hauptergebnis betrifft die als modellspezifisch beschriebenen Versorgungsmerkmale: 7 dieser 11 Merkmale zeigen einen starken Effekt für die Unterschiede zwischen Kliniken mit und ohne MV. Ein Nebenbefund ist, dass bei MV mit Regionalbudget die Unterschiede gegenüber der Regelversorgung deutlich stärker ausgeprägt sind. Zwar lässt sich die Umsetzung der Merkmale auch an Kliniken der Regelversorgung nachweisen, jedoch in signifikant geringerem Umfang als in den MV.

Die Merkmale *Flexibilität im Settingwechsel* und *settingübergreifende therapeutische Gruppenangebote* zeigen einen besonders hohen Umsetzungsgrad in den MV. Dies spiegelt sich auch in den Leistungsdaten wider, wonach Patienten aus MV in einer Behandlungsepisode gehäuft mehrere Settings in Anspruch nehmen, öfter zwischen den Settings wechseln und dennoch in der Summe weniger Tage vollstationär behandelt werden.

Dass die *settingübergreifende Behandlerkontinuität* ein Schlüsselmerkmal der MV darstellt, konnte bereits in verschiedenen Studien gezeigt werden [11, 14]. Die vorliegenden Daten machen deutlich, dass auch einzelne Kliniken der Regelversorgung Patienten in verschiedenen Settings durch dieselben Teams behandeln, wenn auch in geringerem Umfang.

Das Merkmal *Zuhausebehandlung* wird von den MV in signifikant höherem Ausmaß umgesetzt [25]. Die Leistungsdaten zeigen, dass zusammengenommen weniger als 1 % der Patienten der Kontrollkliniken aufsuchend behandelt wurden. Dies fügt sich zu den Daten des Deutschen Krankenhausinstitutes, wonach im Jahr 2019 ein äußerst geringer Teil aller psychiatrischen Kliniken eine Form der aufsuchenden Akutbehandlung anbot [26]. Mit fortschreitender Einführung der Stationsäquivalenten Behandlung (StäB) werden die Kriterien des Merkmals *Zuhausebehandlung* jedoch zunehmend von Kliniken der Regelversorgung erfüllt. Um die Unterschiede zwischen den beiden Formen aufsuchender Versorgung künftig trennscharf zu erfassen, sollte die Definition des Merkmals *Zuhausebehandlung* präzisiert werden: StäB erfordert per Definition einen täglichen Patientenkontakt; in der *Zuhausebehandlung* der MV lässt sich die Behandlungsintensität individuell steuern [27]. Dieser und weitere Unterschiede zwischen den Versorgungsformen sind bei der kontinuierlichen Weiterentwicklung der Merkmale zu berücksichtigen.

Die Hypothese, dass jene MV, die einen Modellvertrag über ihr gesamtes Budget vereinbart haben, eine besonders starke Umsetzung der Merkmale aufweisen [14, 17], wird auch durch die vorliegenden Befunde gestützt. Eine mögliche Erklärung hierfür liefert ein prozessevaluatives Teilprojekt der PsychCare-Studie [28]: Demnach könne sich in diesen Kliniken vollständig auf die Entwicklung und den Betrieb der Modellversorgung fokussiert werden, während jene selektivvertraglichen MV durch den simultanen Betrieb der Regelversorgung bei der Umsetzung der Modellmerkmale limitiert würden. Inwiefern dieser oder weitere Kontextfaktoren, wie z. B. Vorerfahrungen mit ähnlichen Versorgungsmodellen wie dem RPB nach § 24 BPflV, den Umsetzungsgrad beeinflussen, bedarf weiterer Untersuchungen.

Im Gruppenvergleich der Modell- und der Kontrollkliniken fällt auf, dass die Standardabweichung des Implementierungsgrades in den MV überwiegend höher als in den Kliniken der Regelversorgung ist. Dies könnte mit der hohen Heterogenität der MV erklärt werden: Abgesehen von den gesetzlichen Vorgaben, eine „sektorenübergreifende Leistungserbringung […], einschließlich der komplexen psychiatrischen Behandlung im häuslichen Umfeld“ anzubieten [4], werden die Schwerpunkte jedes MV individuell auf Grundlage struktureller Besonderheiten der Versorgungsregion und Klinik vor Ort abgestimmt. Darüber hinaus werden Steuerungsziele, wie z. B. eine Ambulantisierungsquote mit den Kostenträgern vereinbart. Es verwundert demnach nicht, dass die Merkmale an den einzelnen MV unterschiedlich stark ausgeprägt sind.

Mit Blick auf eine potenzielle Verstetigung der Modellversorgung oder einzelner Versorgungsbestandteile gewinnt die Weiterentwicklung der Merkmale hin zu Qualitätsindikatoren an Relevanz. Derzeit werden in einem weiteren Studienteil der PsychCare-Studie Indikatoren für eine patientenzentrierte und sektorenübergreifende psychiatrische Versorgung entwickelt [19]. Ein Manual sowie das (Core‑)Indikatorenset liegen bereits vor und werden in Kürze publiziert.

### Stärken und Limitationen

Die vorliegende Arbeit ist die erste vergleichende Untersuchung von Versorgungsmerkmalen settingübergreifender psychiatrischer Behandlung an Kliniken der Regel- und der Modellversorgung.

Eine mögliche Einschränkung besteht darin, dass die Leistungsdaten der Studienzentren jeweils pauschal und nicht auf Einzelfallebene übermittelt wurden. Trotz einer umfassenden Verfahrensanweisung konnte die korrekte Datenerhebung an den einzelnen Zentren nicht in jedem Einzelschritt nachvollzogen werden. Die gelieferten Informationen wurden jedoch einem umfassenden Plausibilitätscheck unterzogen.

Neben dem *Umfang der Krankenkassenverträge* stellt die *Dauer der Umsetzung* der MV, einschließlich möglicher Vorerfahrungen mit vergleichbaren Versorgungformen (z. B. RPB), eine weitere potenzielle Einflussgröße des Implementierungsgrades der modellspezifischen Merkmale dar. Letztere konnte jedoch nicht berücksichtigt werden, da nicht ausreichend Fälle (hier: Kliniken mit MV) für eine multivariate Analyse zur Verfügung standen. Die Durchführung einer separaten bivariaten Analyse für die Variable *Dauer der Umsetzung* hätte wiederum zu verschiedenen Effektgrößen geführt. Aufgrund eines nicht auszuschließenden Bias durch unberücksichtigte Einflussgrößen des Implementierungsgrades der MV sollten die Ergebnisse der Subgruppenanalyse vorsichtig interpretiert werden.

An der vorliegenden Studie nahmen nur 9 von insgesamt 22 derzeit bestehenden MV teil. Dies schränkt die Übertragbarkeit der Ergebnisse auf die Gesamtmenge aller MV ein. Dem ist entgegenzuhalten, dass die Studienkliniken so ausgewählt wurden, dass sie ein größtmögliches Spektrum an MV repräsentieren [23].

## Fazit für die Praxis

- Die Struktur- und Prozessmerkmale der Modellversorgung sind geeignet, um Qualitätsunterschiede bei der Umsetzung flexibler und integrativer psychiatrischer Behandlung abzubilden.
- Die Merkmale zeigen einen starken Effekt für die Unterschiede zwischen Modell- und Regelversorgung, welche bei Kliniken mit Regionalbudget (Modellvertrag mit allen Krankenkassen) noch stärker ausgeprägt sind.
- Für eine Beurteilung der Qualitätsunterschiede settingübergreifender Behandlung in der Regel- und Modellversorgung auf Bundesebene sollten die Merkmale an einer größeren Stichprobe untersucht werden.

## Footnotes

1 In der englischsprachigen Literatur wird dieses Versorgungsmodell unter dem Akronym „FIT“ bzw. „FIT64b“ („flexible and integrative treatment“) geführt.
